# Nuclear staining and relative distance for quantifying epidermal differentiation in biomarker expression profiling

**DOI:** 10.1186/1471-2105-9-473

**Published:** 2008-11-06

**Authors:** Thora Pommerencke, Thorsten Steinberg, Hartmut Dickhaus, Pascal Tomakidi, Niels Grabe

**Affiliations:** 1 Hamamatsu Tissue Imaging and Analysis (TIGA) Center, BIOQUANT, University Heidelberg, BQ0010, Im Neuenheimer Feld 267, 69120 Heidelberg, Germany; 2 Institute for Medical Biometry and Informatics, University Hospital Heidelberg, Im Neuenheimer Feld 305, 69120 Heidelberg, Germany; 3 Department of Orthodontics and Dentofacial Orthopedics, Dental School University of Heidelberg, Im Neuenheimer Feld 400, 69120 Heidelberg, Germany

## Abstract

**Background:**

The epidermal physiology results from a complex regulated homeostasis of keratinocyte proliferation, differentiation and death and is tightly regulated by a specific protein expression during cellular maturation. Cellular *in silico *models are considered a promising and inevitable tool for the understanding of this complex system. Hence, we need to incorporate the information of the differentiation dependent protein expression in cell based systems biological models of tissue homeostasis. Such methods require measuring tissue differentiation quantitatively while correlating it with biomarker expression intensities.

**Results:**

Differentiation of a keratinocyte is characterized by its continuously changing morphology concomitant with its movement from the basal layer to the surface, leading to a decreased average nuclei density throughout the tissue. Based thereon, we designed and evaluated three different mathematical measures (nuclei based, distance based, and joint approach) for quantifying differentiation in epidermal keratinocytes. We integrated them with an immunofluorescent staining and image analysis method for tissue sections, automatically quantifying epidermal differentiation and measuring the corresponding expression of biomarkers. When studying five well-known differentiation related biomarkers in an epidermal neck sample only the resulting biomarker profiles incorporating the relative distance information of cells to the tissue borders (distance based and joint approach) provided a high-resolution view on the whole process of keratinocyte differentiation. By contrast, the inverse nuclei density approach led to an increased resolution at early but heavily decreased resolution at late differentiation. This effect results from the heavy non-linear decay of DAPI intensity per area, probably caused by cytoplasmic growth and chromatin decondensation. In the joint approach this effect could be compensated again by incorporating distance information.

**Conclusion:**

We suppose that key mechanisms regulating tissue homeostasis probably depend more on distance information rather than on nuclei reorganization. Concluding, the distance approach appears well suited for comprehensively observing keratinocyte differentiation.

## Background

Epidermal homeostasis is the complex regulated internal equilibrium of cell proliferation, cell differentiation and cell death leading to the constant self-renewal of the tissue. Currently, only few systems biological models, describing aspects of epidermal, or more general epithelial tissue homeostasis have been published [1-4]. Computational physiological models like those of the heart [5] are well known and widely regarded as being fundamental to a real understanding of the functions of tissues and organs. For computational modelling of epidermal or even epithelial tissue homeostasis in general, multi-scale models simulating genetic networks embedded in multi-cellular models are to be expected to emerge in the near future. For any of those models quantitative information will be pivotal. This latter information is to specify how the spatial expression patterns of relevant biomarkers correlate with cellular differentiation throughout the full life time of cells in the tissue.

Cellular differentiation begins at the individual stem cell. Fundamental aspects of the biology of stem cells in skin have been revealed in the last decades [6,7]. The basal compartment of the epidermis is considered to contain stem cell-like cells as well as early differentiated cells. Cells leaving this compartment are subject to a complex molecular process called terminal differentiation leading to the formation of the cornified envelope. Although being continuous, this process has so far been described only in terms of qualitative milestones like keratin K1/10-, involucrin-, and filaggrin-expression [8]. In literature, a quantitative model of epidermal differentiation as a continuous process does currently not exist. Relevant technologies for this task could be based on gene expression arrays, which have been used to reveal general building blocks of epidermal differentiation [9,10] or tissue profiling like mass spectrometry [11].

Immunofluorescent histological tissue sections represent a well suited, reliable, time-, and cost-effective means for assessing structural and functional aspects of tissues including differentiation. In stained sections of stratified epithelial tissues, the topographical gene and protein expression patterns are directly linked to the respective position of cells in the tissue. While the cells gradually change their position in the tissue, they differentiate and accordingly alter their molecular composition at the mRNA and protein level. Therefore, observing topographic biomarker expression patterns of stratified epithelia in tissue sections principally allows the measurement of the average changes of protein expression during cellular differentiation. For systems biology, such topographic expression changes are highly interesting since they facilitate the observation of the biological consequences of the temporal mRNA and protein networks regulating cellular differentiation from stem cell-like cells up to terminally differentiated cells. Recently, we demonstrated how, principally, such temporal networks can be reconstructed from histological sections at the example of human epidermis [12].

**Figure 1:**
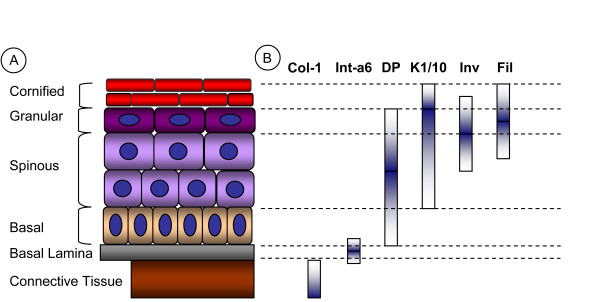
**Biomarkers of epidermal differentiation**. (A) Layer of human epidermis arising from skin differentiation. Increasing distance from the connective tissue CT is correlated with increasing differentiation. (B) Schematic protein biomarker expression patterns. Col-1 = collagen-1, Int-a6 = integrin alpha 6, DP = desmoplakin, K1/10 = keratin K1/10, Inv = involucrin, Fil = filaggrin.

First qualitative assessments of epidermal differentiation using immunohistology have been reported early [13]. Selected quantitative studies on changes in epidermal histology [14-16], or epidermal nuclei [17,18] have been described in the early 90s. Quantitative measurements of the spatial distributions of biomarkers in epithelial tissues, however, are still missing in literature [8]. According to the best of our knowledge, studies of well known differentiation biomarkers of skin like involucrin [19], keratin 1/10 [20,21], desmoplakin [22,23], integrin-α6 and filaggrin [8] have not yet been correlated with a quantified degree of cellular differentiation. The prevalent view on epidermal differentiation is based on the four layers: Stratum basale, Stratum spinosum, Stratum granulosum and the Stratum corneum. These layers are typically determined by their distinct cellular morphology and the expression of specific biomarkers like keratin 1/10 for suprabasal layers and filaggrin for Stratum granulosum. Figure 1 shows the expression of a representative and commonly used set of biomarkers throughout the aforementioned epidermal layers.

The present report shows how to each epidermal location in stratified epithelia an average degree of differentiation can be assigned. We approximate this degree using distance information and nuclear stain. The work is based on image processing on digitalized immunofluorescent histological sections of human skin. In the epidermis, cellular differentiation progresses constantly while cells move relatively "upwards" towards the surface of the tissue [24]. Further hallmarks of differentiation are the change in cellular size (cytoplasmic growth), chromatin decondensation and the final degradation of nuclear DNA during corneocyte formation [25]. The differentiation related changes to nuclei can be observed via a general loss of nuclear stain throughout the epidermis towards the tissue surface. Based on these phenomena, we developed two basic hypotheses as to how tissue differentiation can be quantified: (a) the assumption that the relative position of a cell with respect to the tissue borders reflects its level of differentiation suggests to measure differentiation by the relative position of a keratinocyte on its spatial trajectory through the epidermal tissue. This corresponds to a geometric interpretation of the regulation of keratinocyte differentiation. Or (b) from a more functional point of view: the assumption that tissue differentiation is correlated to the mean nuclei density, suggests to estimate differentiation as the mean DAPI intensity per tissue area.

The mean DAPI intensity in the epidermis is probably determined through the functional sequence of the main steps in a keratinocyte's life comprising basal detachment initiating nuclear reorganization, cytoplasmic growth, nuclear degradation and finally corneocyte formation. We implemented these hypotheses in the form of three differentiation measures; namely by calculating either 1.) the relative distance inside the epidermis to the connective tissue (distance approach) or 2.) the average nuclei density (inverse nuclei density approach) or 3.) the equidistant nuclei density, a joint approach combining distance information with measured nuclear staining (equidistant nuclei density approach).

For the evaluation of the three measures of epidermal differentiation we used the principles of a protein biomarker profiling approach described by the authors recently [12]. There, we measured the expression of protein biomarkers against its relative location in the epithelial compartment in epidermal histological sections. In the present work we use this approach to investigate the effect of the three different measures of epidermal differentiation on protein biomarker profiles of epidermal homeostasis. In order to reveal the impact of spatial information on cell differentiation, the profiling of the first approach is solely based on relative distance. By contrast, the second approach is independent of distance information. Finally, the third approach combines the distance information with the DAPI intensity information. After all, we characterize the properties of the three distinct measures and their suitability for biomarker profiling of epidermal homeostasis. Through this, in particular, we expect to gain insights into the spatial regulation of epidermal tissue differentiation.

## Results

### General approach

**Figure 2:**
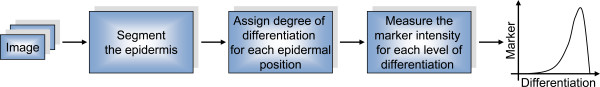
**Schematic illustration of the profiling process**. Image processing of tissue samples. The epidermis is separated from connective tissue by segmentation. For each pixel location in the epidermis, the degree of differentiation is determined by the here presented differentiation measures. Measuring the mean marker intensity with respect to sufficiently small intervals of differentiation produces a quantitative biomarker profile.

The used protein profiling method is based on the image analysis of fluorescently stained serial tissue sections. In each section a fluorescence triple-staining is applied discriminatively marking the connective tissue (Alexa 594 red stained collagen I), the cell nuclei (DAPI blue) and a protein biomarker of interest (Alexa 488 green). During image acquisition the emitted fluorescence signal is split according to wavelength into the RGB channels. This splitting produces three independent images *I*_*Nuc *_(nuclei), *I*_*Marker *_(marker) and *I*_*Con *_(connective tissue). Furthermore, a phase contrast image *I*_*Phc *_is obtained from the section. In phase contrast microscopy, variations in optical density are translated into intensity thus visualizing objects without any staining. The image processing pipeline is depicted in Figure 2. After image acquisition the epidermis is automatically identified (segmented) based on the staining of the cell nuclei and the connective tissue as well as on the phase contrast image. A detailed description and validation of the segmentation algorithm is provided in the methods section.

To determine a two-dimensional profile of protein expression describing keratinocyte's differentiation, the intensity of the biomarker's staining inside the epidermis is measured against the state of differentiation. Here, it is assumed that the strength of protein expression directly correlates with the intensity of the biomarker staining. For the analysis of the average co-expression, serial sections are stained in such a way that in each section a different biomarker is analysed. For each section, i.e. protein, an individual biomarker profile is calculated. The resulting profiles are then superimposed, allowing for the search of related expression patterns among the individual biomarkers. To increase the confidence level of the calculated profiles, multiple sections can be stained repeatedly with the same biomarker. The corresponding profiles for a given protein are averaged, producing a more representative biomarker profile.

### Measures of differentiation

We consider a quantitative differentiation measure to be a function which assigns an approximate degree of cellular differentiation to each location inside the epidermis. To this end, we present three different approaches. The first approach quantifies differentiation via the relative distance to the connective tissue with respect to the epithelial thickness at that position. The second and third approach are nuclei-based measures quantifying differentiation via nuclear staining. The latter depend on the observation that in low differentiated spatial environments cellular nuclei have minimal distances to each other. By contrast, epidermal layers being closer to the surface are characterized by sparsely located nuclei with large distances in between. Therefore, both nuclei-density approaches approximate differentiation by the average nuclei density in the tissue.

### Measuring differentiation by distance

The distance-based measure relies on the assumption that in close distance to the border of the connective tissue epidermal differentiation is minimal (diff = 0%). Differentiation is becoming maximal adjacent to the stratum corneum (diff = 100%). To each position in the epidermis we assign a relative distance to the connective tissue. This relative distance can be interpreted as the relative distance of a differentiating cell on its hypothetical trajectory from the basal layer to the surface of the epidermis. It is calculated as the ratio of the Euclidean distance *d*_*conTis *_of the current pixel to the connective tissue and the length of the shortest path (*d*_*conTis *_+ *d*_*surf*_) from the connective tissue to the surface passing that pixel. This leads to a normalized distance of 0% at the border to the connective tissue and a distance of 100% at the tissue surface, as indicated by equation 1:

(1) $$d_{dist}=\frac{d_{conTis}}{d_{conTis}+d_{surf}}$$

### Measuring differentiation by nuclei

The two remaining measures are related to nuclei density. These are the inverse nuclei density and the equidistant nuclei density. Both are based on the assumption that cellular differentiation is minimal (diff = 0%) where cells are closest to each other and the spatial cytoplasm to nucleus relation is minimal. This implies a high nuclei density. With proceeding cellular differentiation the cytoplasmic proportion of the cell increases. Finally, as the last step in terminal differentiation, nuclear degradation sets in, leading to a decline of nuclei density.

The basic idea of the nuclei-based methods is to assign a mean nuclei density to the vicinity of each epidermal location by means of nuclear stain. This is accomplished by smoothing the actual image of nuclear staining *I*_*Nuc *_into image *S*_*Nuc*. _The resulting intensities of the latter reflect the mean nuclei density of the neighbourhood for each regarded position. A detailed description of the smoothing procedure is given below.

**Figure 3:**
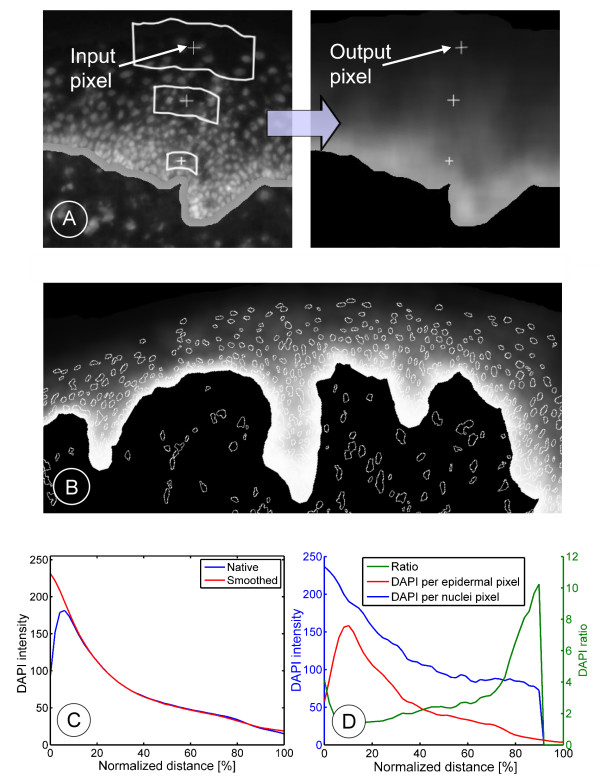
**Smoothing of nuclear staining as used for measuring differentiation**. (A) Illustration of the smoothing process. The band corrected image of nuclear staining is smoothed by averaging over a distance and curvature dependent neighbourhood (filter matrix). In the input and output image three corresponding different epidermal locations are marked, respectively. (B) The mask of all nuclei when superimposed on the smoothed image demonstrates that smoothing removes the original contrast of the nuclei with respect to their environment. (C) Profile of nuclei density measured against the relative distance to connective tissue averaged over a representative set of tissue sections. The blue curve shows the gradient calculated from the original native image of nuclear staining. The red curve is computed on the image after adjusting intensity values in the area of basal lamina and subsequent smoothing. (D) Analysis of the nuclei density in an exemplary tissue sample. The loss of DAPI intensity throughout the complete tissue (blue) results only partly from the loss of DAPI inside each nucleus (red). The ratio of the blue and red graph (green) shows the impact of cytoplasmic growth and nuclear degradation on the loss of DAPI intensity throughout the tissue. At 100% distance to the connective tissue, the loss of DAPI intensity per epidermal area can be attributed almost entirely to cytoplasmic growth and nuclear degradation

Measuring the mean nuclear staining in dependence of the normalized distance to the connective tissue results in the graph of Figure 3C. The diagram shows a function with a maximum at about 5–10% of the normalized distance depending on the thickness of the epidermis, decreasing from that point on. The gradient does not have its maximum at minimal differentiation (diff = 0%), since the underlying epidermal mask includes the epidermal-dermal interaction zone where no nuclear staining is present. However, for measuring the differentiation by nuclei density, we need an unambiguous monotonically decreasing correlation between nuclear stain and the estimated differentiation.

In order to obtain the required monotonically decreasing functional correlation we have modified the intensities in the nuclei image inside a band of half a cell width at the epidermal border to the connective tissue (band-correction). The associated pixels are artificially set to intensity values increasing with the distance to the basal layer. The minimal intensity is chosen to be higher than the mean intensity in the basal layer. In order not to introduce artifacts by smoothing, it is important that the native gradient's shape (Figure 3C) remains constant. Otherwise, the positions of former nuclei could no longer be identified in the smoothed image. Clearly, intensity values after smoothing indicate a mean nuclei density in the tissue and do not belong to a specific nucleus any longer.

Typical smoothing operations use a constant, thus fixed-size and uniform filter throughout the image. A uniform filter assigns the same weight to each filter position. A fixed-size filter determines each value in the smoothed image by averaging for each image pixel over the same amount of neighbouring pixels regardless of the position in the image. Applied to images showing nuclei in epidermal tissue sections such a constant filter could not take into account the enlargement of keratinocytes during differentiation.

Therefore, considering the native gradient of nuclear staining from basal lamina to surface, a distance dependent smoothing algorithm was developed. Here, the image of nuclear staining *I*_*Nuc *_is smoothed by an approximately rectangular filter *N*, whose exact shape is defined by the curvature of the underlying basal lamina. That filter is to be considered a band aligned in parallel to the basal lamina. The length *l*_*p *_as well as the associated height of that band gets enlarged by a factor f proportional to the distance to the basal lamina of the currently considered pixel. The factor f is chosen to roughly reflect the distance between the nuclei starting with about 20 μm (*l*_*min*_) and ending at about 70 μm. These considerations result in equation 2:

(2) $$\begin{array}{c} S_{Nuc}(p)=\frac{\sum_{i=1}^{\left|N(p)\right|}I(N{\left(p\right)}_{i})}{\left|N(p)\right|}\\ N(p)=\{x\in M|M(x)\neq 0\wedge d(x,p)\leq \frac{l_{p}}{2}\wedge \\ \left|d(x,b(x))-d(p,b(p))\right|\leq \frac{l_{p}}{4}\}\\ l_{p}=f\cdot d(p,b(p))+l_{\min} \end{array}$$

with *S*_*Nuc *_the resulting smoothed image and *M *the epidermal mask. *I*(*x*) denotes the value of pixel *x *in the image *I*, being the image of nuclear staining *I*_*Nuc*_. Further, *p *is the current pixel and *N*(*p*) its regarded neighbourhood; *d*(*x*, *p*) is a distance function returning the Euclidean distance between the pixels *x *and *p*; *b*(*p*) returns the closest pixel in connective tissue for the pixel *p*. Finally, *l*_*min *_designates the minimal edge length of the filter matrix. An example of the smoothing process is given in Figure 3A. This smoothing algorithm was applied to a set of nuclei images. The comparison of the gradient of DAPI intensity of the native images with the gradient of the smoothed images (including band correction) discloses that no difference in slope has been introduced by the smoothing algorithm (Figure 3C). To further validate the smoothing algorithm, for each recognized cell nucleus in the native image we determined the contrast to its environment in the smoothed image. We observed no difference in intensity of the nuclear position and its surrounding (exemplarily see Figure 3B). A detailed analysis of the obtained profile of nuclear staining in the context of epidermal differentiation is given in the discussion section (Figure 3D). So far, we have associated a mean nuclei density to each epidermal position. However, the question is, how this density reflected by the intensities in the smoothed nuclei image, can be uniquely mapped onto a biologically interpretable level of differentiation. In the following, we discuss two possible scenarios.

### Inverse nuclei density

In this measure it is assumed that the loss of nuclei occurs linearly during differentiation, meaning that a certain average loss of nuclei always implies the same average increase in differentiation. Letting *d*_*max *_denote the maximal nuclei intensity in smoothed image *S*_*Nuc *_we define

(3) $$diff=\frac{d_{\max}-d_{Nuc}}{d_{\max}}\cdot 100$$

as the mean quantified differentiation. Obviously, this measure is inverse to the nuclei density *d*_*Nuc *_as reflected by the nuclei intensity in the smoothed image.

**Figure 4:**
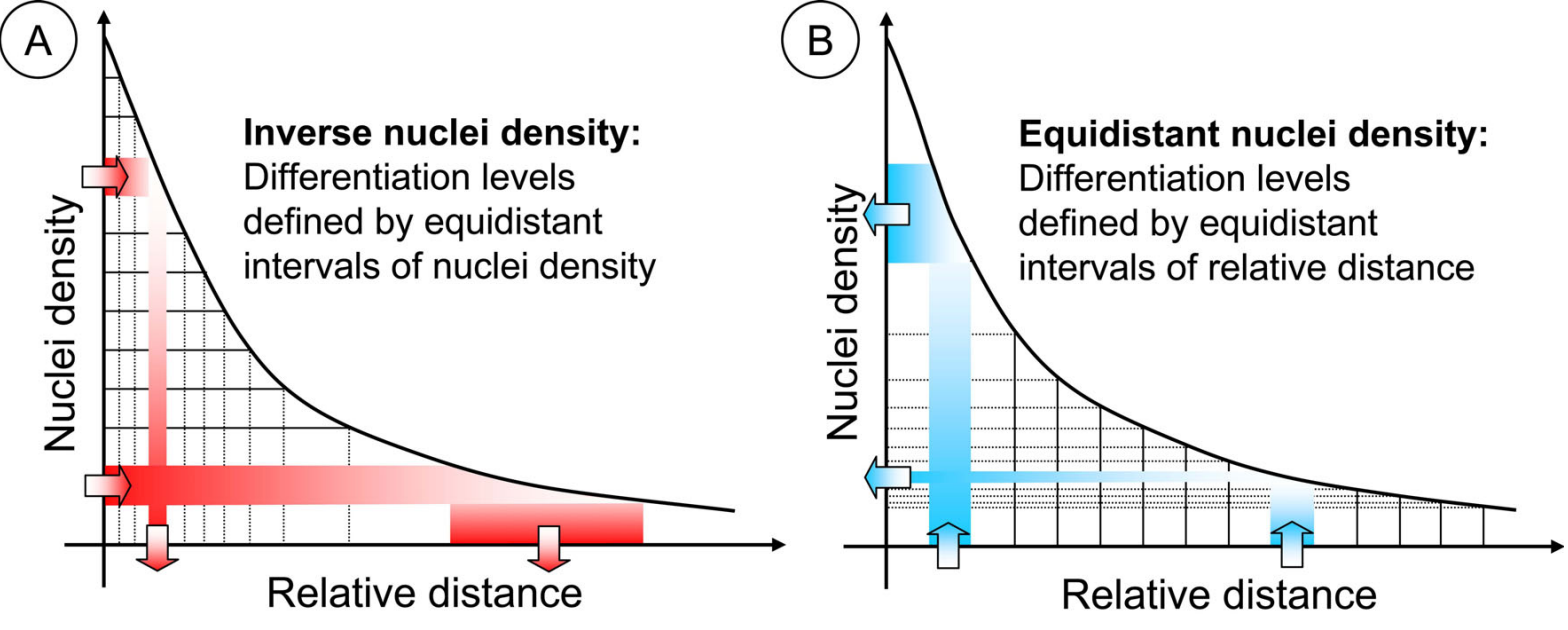
**Relationship between nuclei density and relative distance with respect to both nuclei-based differentiation measures**. (A) Inverse nuclei density approach. Each degree of differentiation is given by an interval of nuclei density of the same width. Intervals of relative distance are compared. Due to the nearly exponentially decrease of the nuclei gradient, an interval of high nuclei density results in a small interval of relative distance. By contrast, an interval of small nuclei density leads to a large interval of relative distance. The equidistant method is illustrated in (B). The degree of differentiation is in the first place defined by the relative distance to the connective tissue. To equidistant intervals, the corresponding intervals of nuclei density are determined. Intervals of small distances lead to large intervals of nuclei density and intervals at larger distances result in narrow intervals of the nuclei density.

The nuclei density is divided into intervals of the same width which are then mapped to the relative distance at which the nuclei densities can be found on the average. We observed that the resulting distance intervals are small at small distances but increase for larger distances (Figure 4A), thereby implying large changes in differentiation at early timepoints of differentiation.

### Equidistant nuclei density

This measure supposes that although nuclei are measured, in the first place differentiation is correlated to the relative distance to the connective tissue. Due to local variances in differentiation, possibly caused by stem cell niches or cutting artifacts, the differentiation might not be directly related to distance. It can be expected, that these local variations in differentiation can be recognized as local alterations in nuclei density. Though we define differentiation intervals by distance, we subsequently map these equidistant intervals to the respective intervals of nuclei density by means of the previously determined nuclei gradient (Figure 3C).

In this way, it is possible to establish a link between nuclei density and differentiation. For each differentiation level corresponding epidermal regions are determined by nuclei density, As shown in Figure 4B the loss of nuclei is very high in the beginning where a small relative distance points to a low differentiation. By contrast, the loss of nuclei decreases while the actual distance increases.

### Comparing the measures by biomarker profiling

By means of the above introduced differentiation measures it is possible to assign a rough quantitative degree of differentiation to each position in the epidermis. During biomarker profiling, both, the local degree of differentiation and the protein expression which is given by the staining intensity, are measured at each location in the epidermis. The profile is generated by plotting the measured average biomarker intensity against the corresponding differentiation.

The profiling process starts with the generation of a reference image in which the intensity of each pixel correlates with the degree of differentiation; in particular with, intervals of quantified differentiation. On the basis of the mentioned reference image, for each possible interval of quantified differentiation corresponding regions in the epidermis are selected. Then we measure the mean intensity of the biomarker in those regions of *I*_*Marker*. _This produces the biomarker intensity belonging to the considered quantitative differentiation interval. Repeating the procedure for all given differentiation intervals leads to a protein expression profile starting with 0% differentiation and ending with 100% differentiation.

To compensate unspecific staining mainly caused by unspecific antibody binding the profiles are background corrected deploying the mean biomarker intensity measured in the connective tissue. Current immunohistochemistry does not provide reliable absolute intensity values. Therefore, after averaging over all quantitative profiles corresponding to one biomarker the intensities of the averaged profile are normalized so that the maximum biomarker intensity found of all differentiation intervals is set to 100. Each of the repeatedly measured profiles is then multiplied by a factor which minimizes the sum of least squares of the average biomarker profile. For each of the corrected profiles, the standard deviation of the mean value is calculated. For co-expression or co-regulation studies, the profiles of different biomarkers are superimposed, provided they are measured by means of the same differentiation measure.

### Measured profiles

**Figure 5:**
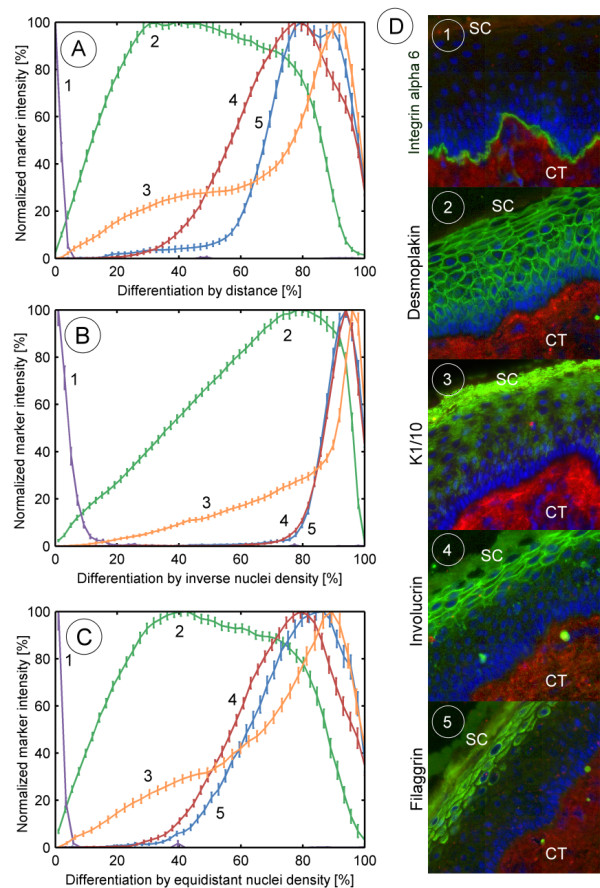
**Multi biomarker profiles for the three differentiation measures**. Comparison of the profiles of the same biomarkers by the (A) distance-based measure, (B) inverse nuclei density-based measure and (C) equidistant nuclei-based measure. In the profiles the individual biomarker are referenced by 1: integrin-α6, 2: desmoplakin, 3: keratin K1/10, 4: involucrin, 5: filaggrin. For each measuring point the standard deviation of the mean is plotted. For a comparison with experimental results an immunohistological staining for each biomarker is presented as well (D). SC = stratum corneum, CT = connective tissue.

To apply the three differentiation measures presented, serial sections of an epidermal neck sample were stained (see the methods section). From these sections, expression profiles of five protein biomarkers (integrin-α6, desmoplakin, K1/10, involucrin, filaggrin) indicating different degrees of differentiation were calculated and superimposed thus producing multi-biomarker profiles. To illustrate the expression patterns, sample images of the immunohistological staining are given in Figure 5D showing the biomarker in green. Integrin-α6 (1), a component of the hemidesmosomes, is visible as a thin line in the basal lamina zone. Desmoplakin (2), a main component of the cell-cell contact mediated by the desmosomes, is expressed in the whole epidermal compartment from the basal layer up to the stratum granulosum.

The expression of K1/10 (3) is switched on in the supra-basal layers, when the composition of keratin-intermediate filaments abruptly changes from K5/14 to K1/10. K1/10 has its main expression level in the stratum corneum. Involucrin (4) is first expressed in late stratum spinosum and thus can be seen as a precursor of terminal differentiation. Involucrin is a substrate of transglutaminase, an enzyme mediating the cross-linking in the corneum. Filaggrin (5) starts shortly after involucrin. It is the main component of the keratinocytes during terminal differentiation, formed through cleavage of its predecessor profilaggrin.

The protein profiles calculated for these biomarkers are shown in Figure 5ABC. In general, we observe a weak, unspecific staining in the epidermis for all biomarkers. Therefore, we consider any of the biomarkers to be expressed if a normalized intensity of 10%, taken as a threshold, is exceeded. In the following, we roughly describe the profiles. The first multi-biomarker-profile illustrates the results of the analysis as calculated by the distance-based differentiation measure (Figure 5A). Integrin-α6 (Figure 5A1) reveals a clear expression from 0–5% differentiation. Desmoplakin (5A2) covers the range of 3% to 93%. K1/10 (5A3) is expressed from 12% on, with maximal expression between 80 and 98%. Involucrin (5A4) is expressed from 38% on, reaching its peak expression at 78%. Filaggrin (5A5) starts later with 58% and has a broader peak from 78% till 93%.

The evaluation of the protein expression by means of the measure of inverse nuclei density shows different profiles (Figure 5B). Obviously, the resolution of the profiles is much higher at the beginning of the profiles than at the end. Therefore, the expression profiles are expanded at early and squeezed at late differentiation: The integrin-α6 (5B1) profile calculated by the inverse nuclei density measure thus shows an expression up to 9% differentiation. The expression of desmoplakin (5B2) increases slowly reaching its peak expression between 55% and 95%. Both involucrin (5B4) and filaggrin (5B5) have their top expression at 95%, and thus have indistinguishable expression profiles. K1/10 (5B3) is fully expressed later at 98%.

The expression profiles for the equidistant nuclei density measure (Figure 5C) resemble very much those of the distance measure, with the exception that the resolution of the profiles changed for the worse. This is particularly noticeable in the expression profile of K1/10 (5C3) not showing the steep increase at 80% differentiation (see Figure 5A, distance measure).

## Discussion

Epidermal homeostasis with its innate differentiation coupled protein expression is far from being fully understood. In particular, quantitative models of this homeostatic process are lacking, although such formalizations are to be expected fundamental for understanding complex disease related processes like wound healing and response to toxic reactions. As a basic approach for investigating tissue homeostasis with the means of systems biology we developed a method for measuring spatial profiles of protein expression in epidermis[12]. Such profiles should provide a quantitative reference data set for single cell based models of tissue homeostasis, which will have to assign a specific level of differentiation to any virtual cell of the tissue. To this end we presented and evaluated three different approaches for the quantification of epidermal tissue differentiation: the distance-based, the inverse nuclei density-based and the equidistant nuclei-based measure. To assess to which extent the developed measures are suitable for characterizing differentiation, we used the latter to study biomarkers of differentiation. The resulting multi-biomarker profiles, obtained by superimposing the individual biomarker profiles (Figure 5ABC), generally agree with the sequence of the differentiation events described in literature (Figure 1) as well as with the microscopic images depicted in Figure 5D. In contrast to the common picture of epidermal tissue differentiation as a process organized mainly in four layers, the measures we here presented are continuous by nature and allow the generation of continuous but nevertheless clearly distinguishable biomarker profiles.

Comparing the produced quantitative profiles it becomes evident that both, distance measure and the equidistant nuclei measure, produce nearly identical profiles, while the inverse nuclei density-based profiles seem to be stretched at early and compressed at higher differentiation. We explain this through a sampling resolution being high at early differentiation and decreasing with progressing differentiation – the latter effect being due to the rapid loss of DAPI intensity at the beginning. This predestines the inverse nuclei density measure for the analysis of early events of epithelial differentiation. However, events of terminal differentiation cannot be distinguished clearly. The inverse nuclei density measure would therefore lead to an unbalanced view on epidermal differentiation. Hence, this measure did not seem to be capable of discriminating the representative set of biomarkers, comprehensively characterizing the whole epidermal differentiation process.

We can conclude that the distance measure and the equidistant nuclei measure produce a much more comprehensive, high-resolution view of differentiation. The equidistant nuclei approach as a combination of both previously discussed measures leads to profiles very similar to those calculated with the distance measure. However, the resolution in later differentiation stages is reduced slightly as the intensities of nuclear staining lie very close together in that part of the nuclear gradient. This makes the analysis very prone to local variations, which at the same time appears to be a potential advantage of this measure. Accordingly, further studies on tissue samples of other epithelia with strong mesenchymal infoldings or other complex 3D structures have to be made in order to estimate the potential benefit of this measure.

In general, the quantitative correlation between keratinocyte differentiation and DAPI intensity is influenced by many factors and has, to our knowledge, not yet been addressed in literature. We therefore performed a more detailed analysis of the quantitative spatial profile of the nuclei's intensities throughout the epidermis. As expected, the profile turned out to be monotonically decreasing from the peak expression in the basal layer (Figure 3C). A main reason for this decline is the cytoplasmic growth during epidermal differentiation. But also the nucleus itself is subject to rearrangements. We therefore further assessed in how far the measured nuclear signal itself depends on keratinocyte differentiation. Correlating the DAPI signal per nucleus with the relative distance to the connective tissue (Figure 3D) we observed an unexpected high loss of the DAPI signal inside each nucleus already in the first half of the tissue followed by a minimal further decay. The loss of nuclear staining occurring during late differentiation can be obviously attributed to nuclear degradation. According to a systematic study of the relation of chromatin condensation and DNA accessing dyes in calf thymocytes [26], which may be of general nature for eucaryotes, we interpreted the strong early loss of the DAPI signal as a strongly reduced chromatin packing ratio (definition see [26]) in keratinocytes leaving the basal layer. Dividing the distance profile of the mean DAPI intensity per nuclei by the distance profile of the mean DAPI intensity per epidermal area, results in a ratio whose increase towards higher differentiation demonstrates the loss of nuclei density by cytoplasmic growth and nuclear degradation in epidermal differentiation. As the DAPI intensity per nuclei decays only minimally in the upper second half of the epidermis and the before mentioned ratio increases strongly, we concluded that in final differentiation, the loss of DAPI signal is to be attributed almost entirely to cytoplasmic growth and nuclear degradation.

The distance based measure suggests a continuous differentiation with a linear dependency between distance and differentiation. However, the layered architecture of epidermal differentiation is not directly reflected via the distance based measure. For example, two epidermal samples of quite different thickness will naturally have a basal layer of the same thickness although the width of their stratum spinosums will differ considerably. If, for instance, in one sample the spinosum represents a larger proportion of the epidermis, consequently also the spatial differentiation profile changes accordingly. Considering either thick or thin samples, 90% measured differentiation might point to different degrees of biological differentiation. This is why profiles calculated via the distance measure generally depend on the thickness of the tissue. By contrast, the inverse nuclei density approach is almost independent of the thickness of differentiation layers due to the direct mapping of nuclei density and differentiation.

Irrespective of the chosen measure, the profiles obtained by our method necessarily suffer from a certain limit of resolution. For example, although integrin-α6 is visible as a very narrow band in the immunofluorescent images, this band appears in the form of a statistical distribution in the quantitative profile. Similarly, in the histological image of keratin K1/10 the associated expression is characterized by a very sharp switch at the basal-suprabasal transition, which is visible in our profiles as a specific distribution. Also the DAPI profile measured in Figure 3C displays at its beginning a statistical distribution instead of a step-function which would have been expected from the band-like DAPI intensity in the basal layer. This distribution in the profile illustrates the general limit of resolution of the profiling.

The attainable resolution of all biomarker distributions depends on the segmentation algorithm, the choice of a certain differentiation measure and the type and thickness of the epidermis investigated. For quantifying the error introduced through the segmentation algorithm we compared the results of the manual segmentation with our automatically obtained results. We calculated a small deviation of 2.3 μm on the average, to this extent limiting the resolution of the profiles.

In this work, we analyzed three basic quantitative measures of differentiation. The answer to the question which measure should be chosen depends on whether either a specific window of differentiation or whole differentiation is to be studied. While the inverse nuclei density approach seems advantageous for the analysis of early differentiation events, the equidistant nuclei approach appears suggestive for samples having a very irregular structure. The distance method seems to be a good general purpose approach, although the resulting quantitative profiles have to be interpreted in terms of the thickness of the studied tissue. In case of studying tissue samples of different thickness, we therefore recommend not to liken the resulting profiles directly, but rather compare the sequence of the main changes or peaks in the according expression profiles.

Finally, we turn to the question of whether the comparison of the profiles produced by three different measures could provide insight into the regulation of epidermal differentiation. The strong decay of DAPI intensity probably caused by chromatin decondensation and cytoplasmic growth indicates complex reorganizations of the keratinocyte during early differentiation. During further differentiation, the DAPI profile showed a slower monotonous decay, thus indicating a clear correlation to differentiation throughout the whole tissue. On the other hand, late differentiation events could not be profiled with sufficient resolution. Only by adding the distance information to the DAPI intensity based profiling (achieved by the joint measure) it is possible to distinguish the biomarkers depicted in Figure 1. The latter biomarkers are widely accepted as they reflect the common layers of epidermal differentiation. Therefore, we suppose the uneven distribution of nuclei density and intensity to be probably less closely involved in the regulation of tissue differentiation than we originally expected.

## Conclusion

The measures we presented here are continuous by nature and allowed the generation of continuous and distinctive biomarker profiles. Hence, we conclude that our results support the view of differentiation as a continuous process. Moreover, the comparison of the different profiles indicates that the geometrical organization of the tissue has – possibly by key drivers in the form of cell signalling – a close impact on the regulation of protein expression during epidermal differentiation. In the near future we expect to see computational multi-scale models of stratified epithelia like the epidermis with molecular networks embedded in virtual cells characterized by different quantitative levels of differentiation. The methods and data presented here are considered a basis for the tissue differentiation related aspects of this endeavour.

## Methods

### Immunofluorescent staining

The tissue sample analyzed originated from human epidermal neck and was obtained from a healthy patient with informed consent according to the Helsinki Declaration. The protocol was approved by the institutional ethic committee. Sections with a mean thickness of 8 μm are cut from the frozen tissue sample. Staining, immunofluorescent staining, antibody dilutions and imaging have been performed as described previously [12].

### Preprocessing of the images

For each biomarker we analyzed four to six stained sections. As each biomarker profile is calculated from one image the section images were automatically divided into smaller images to increase the statistical sample size. The smaller images showed nearly the same epithelial length of 6 mm. In this way, we were able to carry out a sound statistical evaluation of the biomarker profiling resulting in about 40 profiles for each biomarker, where the previously presented image analysis algorithms were applied. Prior the segmentation of the epidermis, the images were preprocessed starting with a noise reduction by median filtering and the removal of bright interferences, which we attributed to impurity traces caused by sugar. Moreover, on the images used for segmentation (the phase contrast image *I*_*phc*_, the nuclei image *I*_*Nuc *_and the image showing the connective tissue *I*_*conTis*_) an intensity adjustment was performed to enhance the contrast and the robustness of the algorithm

### Segmentation of the epidermis

In a first step in segmentation, tissue borders are identified by edge detection in the phase contrast image *I*_*phc*_. After this, the tissue area is then determined by several morphological image processing operations like dilation and erosion on the edge image. To separate the epidermis from the connective tissue, the basal lamina is determined by edge detection in the image *I*_*Nuc *_of nuclear staining. For edge detection, a Canny method is used specially parameterized for the detection of long stretched edges. By thresholding with hysteresis, put into effect by two deviant thresholds, weak edges are automatically hooked up to previously detected strong edges. This proceeding avoids breaking up of the edge contour caused by noise. However, larger disturbances like those caused by several missing nuclei in a line cannot be overcome by this method. Therefore such gaps occurring in the edge contour are artificially closed by connecting endpoints of nearby edge segments.

The resulting preliminary basal lamina occasionally shows artifacts like false edges and connections, producing a border which separates the tissue area not only into the two regions epidermis and the connective tissue, but in a set of regions. Generally, regions belonging to the epidermis are characterized by a low collagen-I staining being lowest in the direct neighbourhood of the determined basal lamina. Therefore, those tissue regions are assigned to the epidermis which have a low mean collagen intensity close to the determined basal lamina. In a next step islands of connective tissue inside the epidermis eventually produced by cut dermal infoldings are excluded from the determined epidermal area. These islands are identified by a strong collagen type-I staining.

For the subsequent profiling, we need to determine which pixel of the epidermal contour belongs to the surface (the apical side of the epidermis) and which to the basal compartment.

**Figure 6:**
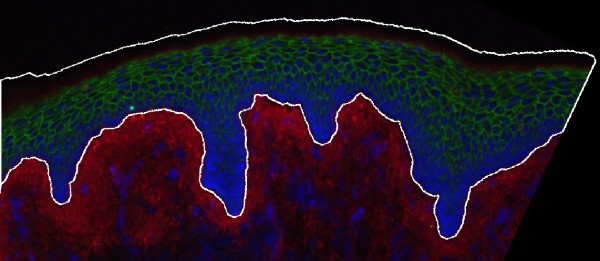
**Example of an automatic segmentation of the epidermis**. The automatically determined contour of the epidermal compartment in the tissue sample is drawn as a white line.

Computationally, we identify the basal lamina as being those contour pieces that have a high nuclear staining in their neighbourhood. Gaps are closed by walking along the epidermal contour from one piece to the next. The surface is identified by region growing of the determined basal lamina towards the tissue surface. Finally, the lower border of the epidermis detected so far results from the last stained line of cell nuclei. To account for the cytoplasmic compartment and the hemi-desmosomal epidermal-dermal interaction zone, we shifted this line by half of a cell diameter beyond that border into the connective tissue. We validated the segmentation algorithm outlined here by comparing the results from our automatic segmentation with those from manually delineated epidermis. To accomplish this test, epidermal tissue of the neck was examined. 46 images from different sections were analyzed each of them manually as well as automatically. A representative example of the automatically determined epidermal contour is shown in Figure 6. We achieved a mean Euclidian distance of 2.3 μm between the manually and the automatically determined contour. For our purposes this indicates a sufficient segmentation accuracy.

All algorithms for image analysis were implemented using MATLAB 7.5 including the image processing toolbox.

## Authors' contributions

TP performed the image acquisition, the algorithm development for image analysis, the interpretation of data and was involved in the conception of the experiments. PT and TS performed the experiments and contributed reagents and materials. HD contributed materials and analysis tools. NG conceived and performed the experiments and interpreted the results. TP and NG wrote the manuscript. All authors were involved in valuable discussions and critically revised the manuscript.
